# Standard method for microCT-based additive manufacturing quality control 2: Density measurement

**DOI:** 10.1016/j.mex.2018.09.006

**Published:** 2018-09-26

**Authors:** Anton du Plessis, Philip Sperling, Andre Beerlink, Lerato Tshabalala, Shaik Hoosain, Ntombi Mathe, Stephan G. le Roux

**Affiliations:** a CT Scanner Facility, Stellenbosch University, Stellenbosch, South Africa; b YXLON International GmbH, Hamburg, Germany; c National Laser Centre, Council for Scientific and Industrial Research, South Africa

**Keywords:** Standard method for microCT-based additive manufacturing quality control 2: density measurement, Additive manufacturing, MicroCT, X-rayTomography, Non-destructive testing, Standardization, Density

## Abstract

MicroCT is best known for its ability to detect and quantify porosity or defects, and to visualize its 3D distribution. However, it is also possible to obtain accurate volumetric measurements from parts – this can be used in combination with the part mass to provide a good measure of its average density. The advantage of this density-measurement method is the ability to combine the density measurement with visualization and other microCT analyses of the same sample. These other analyses may include detailed porosity or void analysis (size and distribution) and roughness assessment, obtainable with the same scan data. Simple imaging of the interior of the sample allows the detection of unconsolidated powder, open porosity to the surface or the presence of inclusions. The CT density method presented here makes use of a 10 mm cube sample and a simple data analysis workflow, facilitating standardization of the method. A laboratory microCT scanner is required at 15 μm voxel size, suitable software to allow sub-voxel precise edge determination of the scanned sample and hence an accurate total volume measurement, and a scale with accuracy to 3 digits.

• MicroCT-based mean density measurement method.
• Accurate volume measurement and scale mass.
• 10 mm cube sample allows standardization and automation of workflow.

## Graphical abstract

**Figure:**
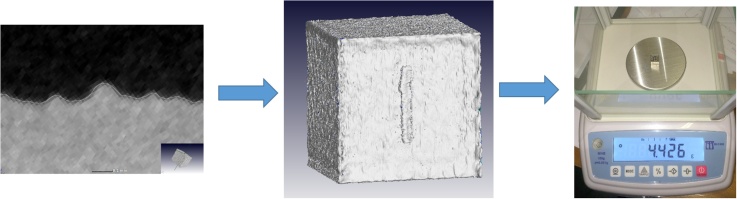

**Specifications Table**

**Table:** 

Subject area	*Engineering*
More specific subject area	*Additive manufacturing*
Method name	*Standard method for microCT-based additive manufacturing quality control 2: density measurement*
Name and reference of original method	*This is a new method, and it is mentioned in my recent review paper: du Plessis, Anton, Igor Yadroitsev, Ina Yadroitsava, and Stephan G. Le Roux. "X-Ray Microcomputed Tomography in Additive Manufacturing: A Review of the Current Technology and Applications." 3D Printing and Additive Manufacturing(2018).It is also used in a recently published round robin test: du Plessis, Anton, and Stephan G. le Roux. "Standardized X-ray tomography testing of additively manufactured parts: a round robin test." Additive Manufacturing (2018).*
Resource availability	This is all in the methodsX paper, including supplementary video

## Method details

Additive manufacturing is a fast growing manufacturing method especially useful for medical and aerospace applications, due to the complexity of design that is possible. Various efforts at qualification of systems and materials for different applications are in process, such as demonstrated for medical applications in [1]. There is a need for standardization in quality inspections especially for microCT but more generally for all NDT methods [2].

Part density is usually measured by the Archimedes method [3], which is a standardized and very accurate method. However, this method does have some drawbacks: it requires an assumption of the parent material density, which can potentially be incorrect for alloys depending on the content of different atomic elements. The second drawback is the possibility for surface roughness to capture bubbles and hence measure a higher volume than expected, while small channels connected to the surface may lead into larger cavities, where the water (or gas) will penetrate the sample and hence a smaller volume recorded, and this may not be visible to the human eye. Unconsolidated powder trapped inside cavities or pores will also result in lower density by Archimedes but not significantly so (due to the mass of the powder), and the presence of a large cavity with unconsolidated powder may be missed or not realized.

While the use of microCT for analysis of porosity is well known (see for example [4]), it is not so well known that accurate material volumes can be determined, which can be used indirectly to calculate a mean density for samples. This is an entirely different approach than measuring porosity, and can be useful when some pores may be smaller than the voxel size, or when the alloy density might be incorrect.

In this work we demonstrate a simple methodology where no sample preparation is required – a simple 10 mm coupon sample (cube) is required. The scan method and analysis workflow makes use of commercial hardware and software, and the steps do not involve any form of human judgement. Such simplified unbiased methods are important to the proper use of the technology to support the additive manufacturing community, and is one of a number of standardized methods developed in our group and mentioned in a recent review of the technology applied to AM [5].

## The method

The samples were built on a custom built selective laser melting platform within a commercial LENS enclosure. The laser used was an IPG YLS 5000 ytterbium 5 kW fibre laser, wavelength of 1076 nm with a delivery fibre core diameter of 50 μm. The scanner used was an Intelliweld 30 FC V system. Materials used were Ti6Al4V provided by TLS Technik GmbH, gas atomized with particle size 20–60 μm. The base plate material is Ti6Al4V 150 mm in diameter approximately 40 mm thick. The hatch parameters used were a power of 3 kW, speed of 3 m/s, and a 240 μm spot size. The contour scans used a laser power of 1 kW where the distance between the hatch and contour and the speed was varied to investigate its effect on density (porosity) and surface finish.

A standard coupon sample of 10 × 10 × 10 mm is suggested for this test. This size allows a reasonably high scan resolution (15 μm) while allowing a large enough sample size for practical purposes. All scanning and image analysis steps are described and thereby standardized, and importantly, none of these depend on human selection and all bias is therefore removed. This method can be cost effective considering the additional information obtained visually regarding the root cause of density variations such as porosity or unconsolidated powder.

The sample is loaded in foam at an angle to ensure no edge artifacts are present, as shown in the first method paper of this series [6]. The method described here therefore does not require a new scan if porosity analysis was already performed as in the above mentioned method, making the mean density measurement simple and fast. The porosity analysis method uses a segmentation process which is almost free of human bias. In the present microCT-density method, the edge of the cube is segmented which is entirely free of human bias and can be fully automated. MicroCT is performed using a standard laboratory microCT system [7], with parameters optimized according to the guidelines presented in [8]. MicroCT scan settings of 200 kV, 70 uA, with 0.5 mm beam filter are used, with image acquisition of 500 ms per image, 2400 step positions in a full 360 ° rotation. At each step position, the first image is discarded and two subsequent images averaged. The total scan time is just under 1 h. When sample setup, machine warmup, background correction and reconstruction is included this should be possible in almost any system in 2 h total. The reconstruction is done using a strong beam hardening correction factor without any image de-noising.

The accurate surface determination is identical to the first part of the workflow in [6] such that the total object volume includes all internal pores. The workflow description is repeated here for this step. The data is analysed in Volume Graphics VGStudioMax 3.1. The image processing steps are described here for removing the exterior air from the data set, but including all material and air (closed pores, not open to surface). Despite the complex description, the video associated with this process demonstrates the simplicity of the process. This segmentation is done by first applying a basic “automatic” surface determination, followed by creating a region of interest (ROI) from this surface. This region is then modified by and opening/closing function with a value of +3, which closes up small surface pores. A region growing tool is then used with high tolerance (no effect of grey values) on the air outside the part, while the option is selected for “avoid other visible ROIs”. This selects all exterior air up to the edge of the part as designated by the surface determination and surface closing function. If small noise particles (loose powder for example) are present outside the part, an opening/closing function (+3) can be applied to this region, to remove these from the selection. Inverting this exterior-air selection allows to select the entire part including its internal voids. A new advanced surface determination function is then applied, using this ROI selection as a starting contour. In this way the local optimization is performed on the exterior surface, allowing the best subvoxel precision on the surface location.

**Fig. 1:**
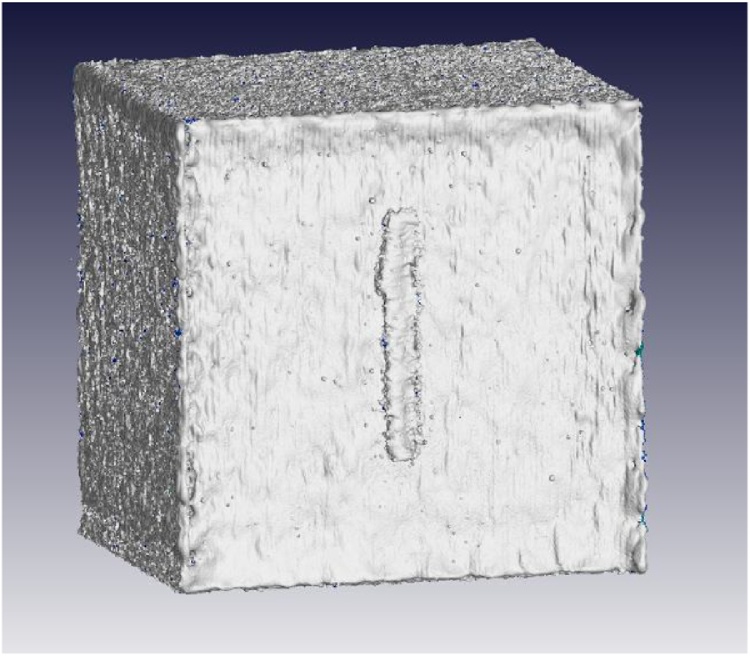
MicroCT image of sample cube with a surface view.

**Fig. 2:**
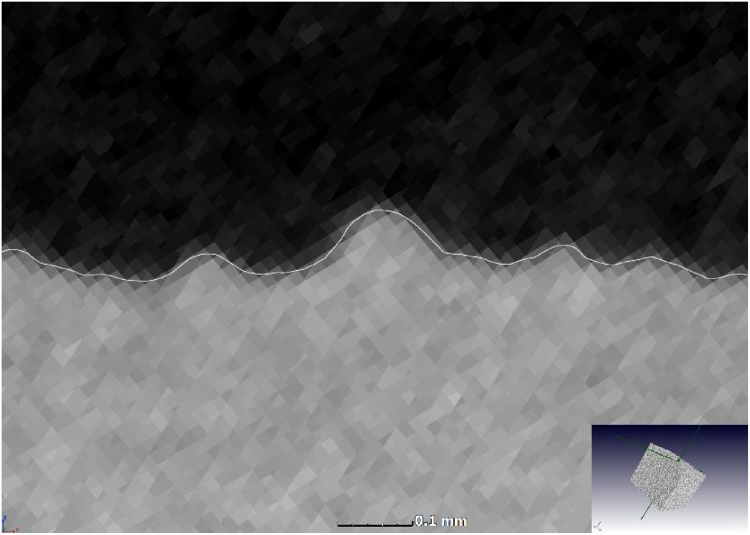
Accurate surface with sub-voxel interpolation shown in close-up view of top surface.

The accurate total volume of the part, including any internal defects is thus found from this object under the properties tab. The above process can be automated and requires no manual input, therefore removing all bias from the results. The resulting visualization of the surface of the sample is shown in Fig. 1. A closeup of one region of the surface is shown in a slice image in Fig. 2, where the white line indicates the location of the sub-voxel precise surface relative to the voxels.

**Fig. 3:**
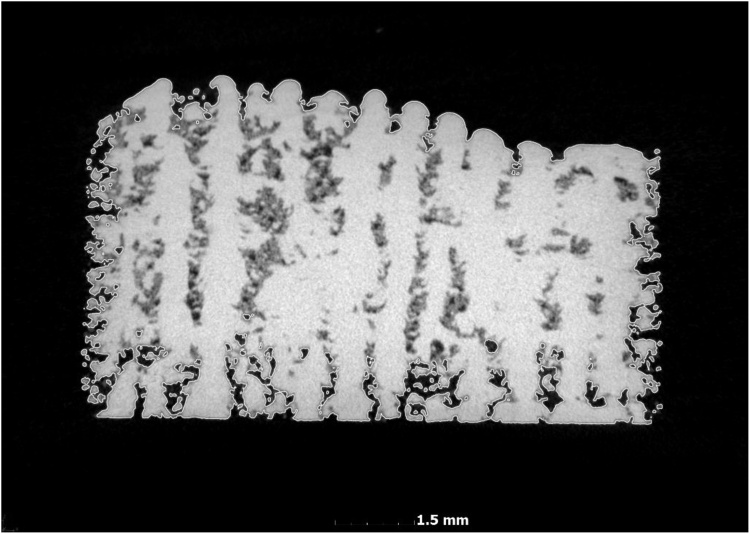
Open and closed porosity in a sample, CT density is calculated from the volume including closed porosity. Seen here is a part with large areas of unconsolidated powder and voids.

It must be noted that large open pores to the surface will be seen as exterior, the same as in an Archimedes test. It is possible to make a different segmentation to include these open pores, but it is not possible to automate this method, as open pores can have significantly varying neck sizes at the surface and the depth of open pores and varying surface roughness will strongly affect results. Therefore this standard method makes use of only the automated steps above. Fig. 3 shows an example of another sample under non-ideal process parameters which contains open and closed porosity: the CT density is only calculated from the material with closed porosity in this standard method.

**Fig. 4:**
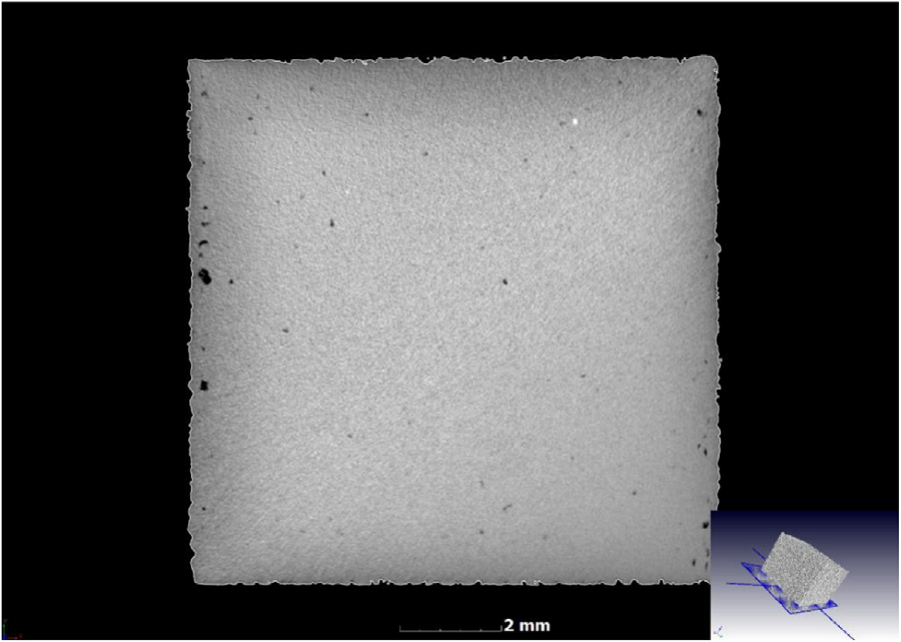
CT slice image shows presence of porosity (black areas) and inclusions (white spots).

The CT density is accompanied by images, which can be used to assess the built part qualitatively. In Fig. 4, the presence of denser inclusions (white spots) indicates impurity present in the powder feedstock. The porosity is observed to be mainly located near the edge, and can be related to the contouring used in this case.

**Fig. 5:**
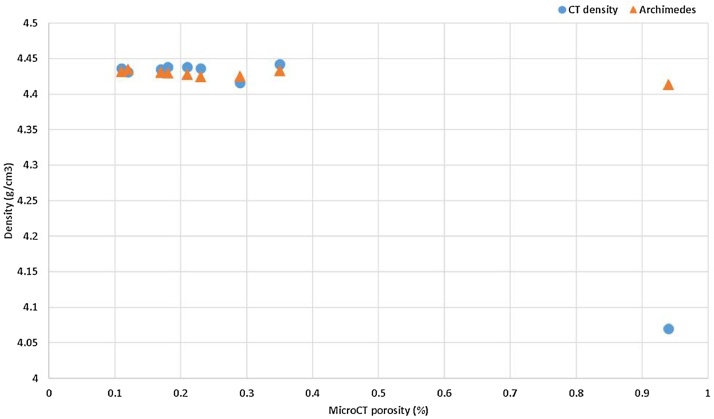
Comparison of CT-density to Archimedes, for a series of 9 coupon samples, relative to traditional microCT porosity measurement. The outlier with high porosity shows that in this case the new CT density method is improved compared to the Archimedes method.

**Fig. 6:**
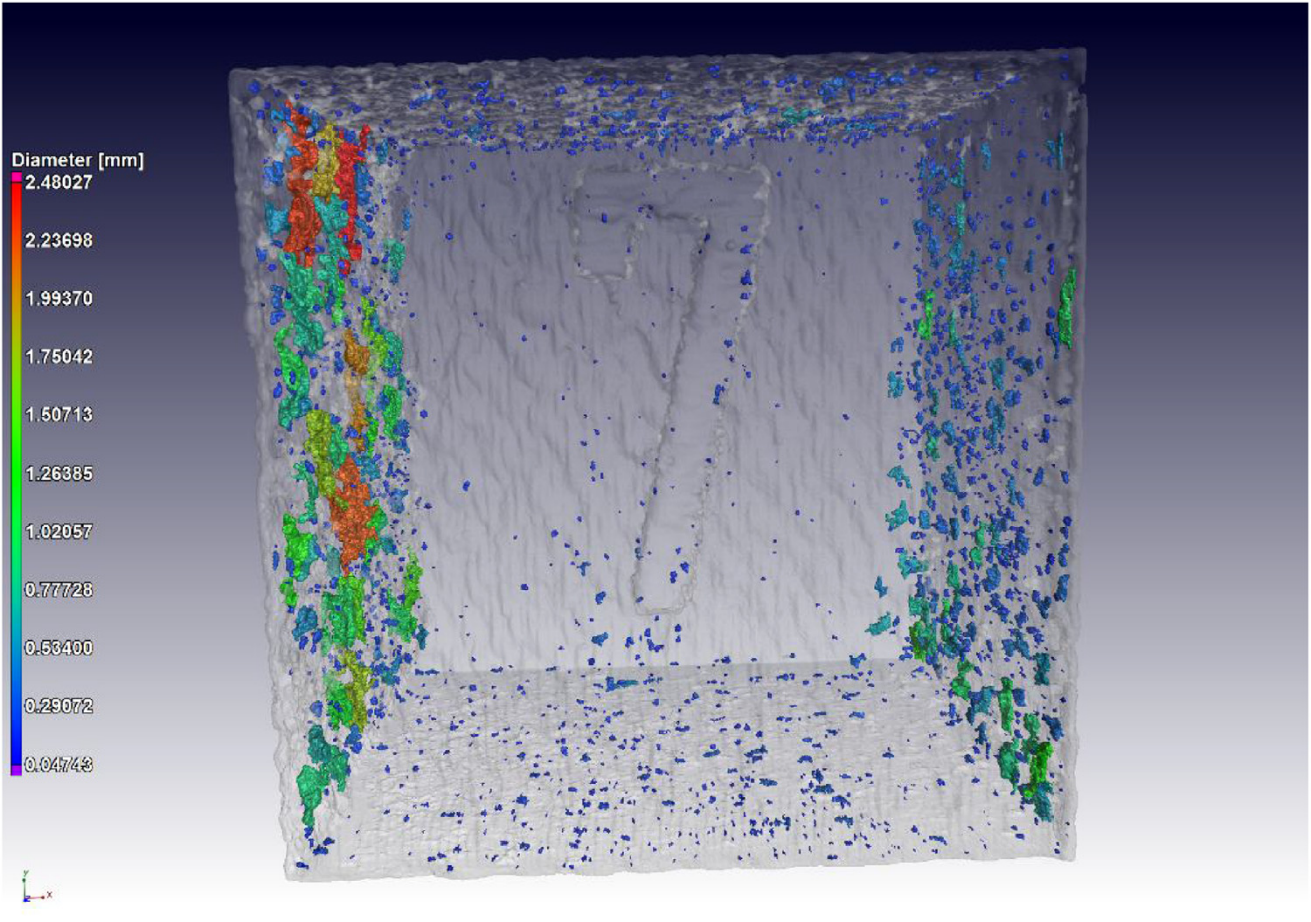
CT image of sample with low CT density, due to large amount of porosity near surface (left in image).

A series of 9 coupon samples were analysed for CT density and traditional Archimedes tests [9] were also done on the same samples: the results compare well, as shown in Fig. 5. Clearly most of the samples were nearly fully dense, except for one outlier. This outlier is shown in a CT image in Fig. 6, indicating the underlying cause of its lower density: large porosity near the surface. The Archimedes method does not work as well as the microCT density method for this outlier, most likely due to connections from surface to the near-surface pores, allowing infiltration of the water into these pores during the Archimedes test. In this series of 9 samples the porosity was not intentionally produced, but can be attributed to increasing scan speed combined with improper contour scanning tracks, not overlapping the filling tracks sufficiently. When the scan speed increases, lack of fusion is more likely to occur and this becomes excessive at the interface between contour and filling tracks.

## Conclusions

The above results show that microCT can be used to calculate an accurate average density from CT-derived volume and scale mass, which is different from traditionally well-known porosity measurement (which may miss small pores, especially smaller than the voxel size). The accurate determination of the sample edge is crucial to the accurate volume measurement and an error margin is expected depending on the surface roughness. Besides surface roughness, dimensional accuracy of microCT systems depends on their regular calibration and some systems are inherently more accurate than others. Using typical laboratory microCT systems, we estimate the error margin for mean density measurement of Ti6Al4V parts of 10 mm diameter to be 0.02 g/cm^3^. In most cases in AM there is more likely to be a larger error in the Archimedes measurement due to rough surfaces trapping gas, open pores allowing water to infiltrate the sample, and unconsolidated powder in the sample which can mask the presence of porosity in the density measurement. This method is likely to play a critical role in optimizing process parameters in the future. The standard methodology described here allows higher throughput and hence lower costs, when using microCT service labs. Since no human input is required, automation in image analysis is possible and can further enhance the throughput for large numbers of identical samples.
